# Calibration Model of a Low-Cost Air Quality Sensor Using an Adaptive Neuro-Fuzzy Inference System

**DOI:** 10.3390/s18124380

**Published:** 2018-12-11

**Authors:** Kemal Maulana Alhasa, Mohd Shahrul Mohd Nadzir, Popoola Olalekan, Mohd Talib Latif, Yusri Yusup, Mohammad Rashed Iqbal Faruque, Fatimah Ahamad, Haris Hafizal Abd. Hamid, Kadaruddin Aiyub, Sawal Hamid Md Ali, Md Firoz Khan, Azizan Abu Samah, Imran Yusuff, Murnira Othman, Tengku Mohd Farid Tengku Hassim, Nor Eliani Ezani

**Affiliations:** 1 Space Science Centre (ANGKASA), Institute of Climate Change, Level 5, Research Complex Building, Universiti Kebangsaan Malaysia, Bangi 43600, Selangor, Malaysia; kemalalhasa@gmail.com (K.M.A.); rashed@ukm.edu.my (M.R.I.F.); 2 School of Environmental and Natural Resource Sciences, Faculty of Science and Technology, Universiti Kebangsaan Malaysia, Bangi 43600, Selangor, Malaysia; talib@ukm.edu.my (M.T.L.); haris@ukm.edu.my (H.H.A.H.); murnira@ukm.edu.my (M.O.); 3 Centre for Tropical System and Climate Change (IKLIM), Institute of Climate Change, Universiti Kebangsaan Malaysia, Bangi 43600, Selangor, Malaysia; fatimah.a@ukm.edu.my (F.A.); mdfiroz.khan@ukm.edu.my (M.F.K.); 4 Centre of Atmospheric Sciences, Chemistry Department, University of Cambridge, Cambridge CB2 1EW, UK; oamp2@cam.ac.uk; 5 Environmental Technology, School of Industrial Technology, Universiti Sains Malaysia, Pulau 11800, Pinang, Malaysia; yusriy@usm.my; 6 Institute for Environmental and Development (LESTARI), Universiti Kebangsaan Malaysia, Bangi 43600, Selangor, Malaysia; 7 School of Social, Development and Environmental Studies, Faculty of Social Sciences and Humanities, Universiti Kebangsaan Malaysia, Bangi 43600, Selangor, Malaysia; kada@ukm.edu.my; 8 Department of Electrical, Electronic and Systems Engineering, Faculty of Engineering and Built Environment, Universiti Kebangsaan Malaysia, Bangi 43600, Selangor, Malaysia; sawal@ukm.edu.my; 9 National Antarctic Research Centre, IPS Building, University Malaya, Kuala Lumpur 50603, Malaysia; azizans@um.edu.my; 10 Nuclear Science Programme, School of Applied Physics, Faculty of Science and Technology, Universiti Kebangsaan Malaysia, Bangi 43600, Selangor, Malaysia; imranyusuff@ukm.edu.my; 11 TXMR Sdn Bhd., No.1, Jalan TS 6/10, Taman Industri Subang, Subang Jaya 47500, Selangor, Malaysia; faridtxmr@gmail.com; 12 Faculty of Medicine and Health Sciences, Universiti Putra Malaysia, Serdang 43400, Selangor Darul Ehsan, Malaysia; elianiezani@upm.edu.my

**Keywords:** air quality monitoring, low-cost sensor, quality control, machine learning

## Abstract

Conventional air quality monitoring systems, such as gas analysers, are commonly used in many developed and developing countries to monitor air quality. However, these techniques have high costs associated with both installation and maintenance. One possible solution to complement these techniques is the application of low-cost air quality sensors (LAQSs), which have the potential to give higher spatial and temporal data of gas pollutants with high precision and accuracy. In this paper, we present DiracSense, a custom-made LAQS that monitors the gas pollutants ozone (O_3_), nitrogen dioxide (NO_2_), and carbon monoxide (CO). The aim of this study is to investigate its performance based on laboratory calibration and field experiments. Several model calibrations were developed to improve the accuracy and performance of the LAQS. Laboratory calibrations were carried out to determine the zero offset and sensitivities of each sensor. The results showed that the sensor performed with a highly linear correlation with the reference instrument with a response-time range from 0.5 to 1.7 min. The performance of several calibration models including a calibrated simple equation and supervised learning algorithms (adaptive neuro-fuzzy inference system or ANFIS and the multilayer feed-forward perceptron or MLP) were compared. The field calibration focused on O_3_ measurements due to the lack of a reference instrument for CO and NO_2_. Combinations of inputs were evaluated during the development of the supervised learning algorithm. The validation results demonstrated that the ANFIS model with four inputs (WE OX, AE OX, T, and NO_2_) had the lowest error in terms of statistical performance and the highest correlation coefficients with respect to the reference instrument (0.8 < r < 0.95). These results suggest that the ANFIS model is promising as a calibration tool since it has the capability to improve the accuracy and performance of the low-cost electrochemical sensor.

## 1. Introduction

Poor air quality has been linked to human health effects with increased associated diseases and symptoms since the rapid growth period associated with the 4th industrial revolution [1,2]. This is partly linked to emissions from combustion processes associated with cheap fossil fuel and coal-based energy. Several pollutants affecting air quality are of major concern in developed countries, especially in urban region, including carbon monoxide (CO), nitrogen dioxide (NO_2_) and secondary pollutants such as ozone (O_3_) [3]. In Malaysia these gas pollutants are usually found at significantly high concentrations over urban areas such as the Klang Valley, as reported by Latif et al., Banan et al., Ahamad et al. and Ismail et al. [4,5,6,7]. In line with these findings, it is important for local authorities to continuously monitor these pollutants.

Typically, air quality monitoring is carried out using a reference technique or equivalent method at a fixed ground location such as chemiluminescent measurements used for NO, NO_2_ monitoring and dispersive infra-red measurements for CO monitoring [8,9]. However, these techniques have been shown to have difficulties in terms of routine maintenance, such as calibration and quality control, in addition to requiring high-security locations to avoid theft [9]. Therefore, there are limited numbers of reference monitoring stations that can provide data and these are generally located away from source emissions. This leads to poor spatial air quality data coverage and the impact of local sources to air quality may not be considered [10]. Thus, alternative air pollution monitoring approaches have emerged such as low-cost air quality measurement techniques [11].

Previous studies have applied low-cost air quality sensor (LAQS) nodes in air quality networks such as at rural and urban sites, road-side sites and also in mobile vehicular measurements [12,13,14]. A large proportion of LAQSs use electrochemical (EC) sensors as detectors to measure several of the common gas pollutants. Compared to the conventional method, LAQSs have brought a new paradigm for air monitoring, making it possible to install sensors in many more locations. However, there is still an issue regarding data quality in sensor applications. Some data sensors are considerably influenced by meteorological conditions such as temperature and humidity, and even interference from other gas air pollutants [15]. In tropical countries such as Malaysia, humidity is higher than in temperate regions and may affect the results from LAQSs. Thus, undertaking LAQS measurements is essential to investigate the performance of these sensors.

Moreover, there is a lack of an established protocol, which is proven to ensure and control the quality of data, especially while LAQSs are deployed in the field [11]. Consequently, methods or algorithms have been developed to solve these obstacles, to observe a linear relationship between the injected known concentration and the corresponding sensor response, temperature and humidity cycle, cross sensitivity with the other gas pollutant. More sophisticated calibration techniques such as the artificial neural network (ANN) techniques are used to correct the raw data [13,16,17,18,19].

ANN methods have previously been applied as tools for modeling nonlinear complex systems and predictions [20]. Several studies in the area of sensor calibration have used an ANN technique. Huyberechts et al. [21] used an ANN to process the signal arising from a three-sensor array for the identification of organic compounds such as methane (CH_4_) and carbon monoxide (CO) at high concentration levels. The results showed that the calibration model which was developed using an ANN had a good quantitative result with a relative error of ≤5%. Multilayer perceptron, one type of structure of ANN, has been used as a tool to analyze data from sensor arrays for the quantification of the concentrations of six indoor air contaminants—formaldehyde, benzene, toluene, ammonia, CO and nitrogen dioxide (NO_2_) [22]. In another study, Spinele et al. [18] proposed several techniques including ANNs and linear/multi-linear regression for the development of a field-calibration model of multiple sensors in order to measure gas air pollutants such as NO_2_, O_3_, CO, carbon dioxide (CO_2_), and nitrogen oxide (NO). They have evaluated and compared each model using data spanning five months from a semi-rural site under varying conditions. The study found that the ANN technique had the best agreement between the sensor and reference instrument compared to the linear and multi-linear regression technique.

Although ANN techniques offer some advantages, they still have limitations, especially in regard to the local minima problem and the difficulty in determining a suitable structure model [23]. Thus, several authors e.g., [24,25] have proposed either a new learning algorithm or have created a new technique to overcome the above limitations and to increase the reliability and accuracy of the ANN. In line with this development, integration between ANNs and the other techniques such as fuzzy logic is possible, leading to a new technique, namely the adaptive neuro fuzzy inference system (ANFIS) [26]. The ANFIS has combined the advantages of both techniques into a single framework. It has the capability to extract information from human expert knowledge as well as data measured into linguistic information automatically and has the ability to adapt with new environmental knowledge, making it convenient for controlling sensors, pattern recognizing and forecasting tasks [26]. The main purpose of this study is motivated by a desire to develop a LAQS system known as DiracSense for surface O_3_, NO_2_ and CO measurement. The sensor will be calibrated using laboratory and field test experiments. Finally, the ANFIS technique is used as the calibration model. In addition, an ANN approach, namely MLP, is used to assess the capability of the ANFIS as the calibration model.

## 2. Methods

### 2.1. DiracSense System

#### 2.1.1. General Overview

**Figure 1:**
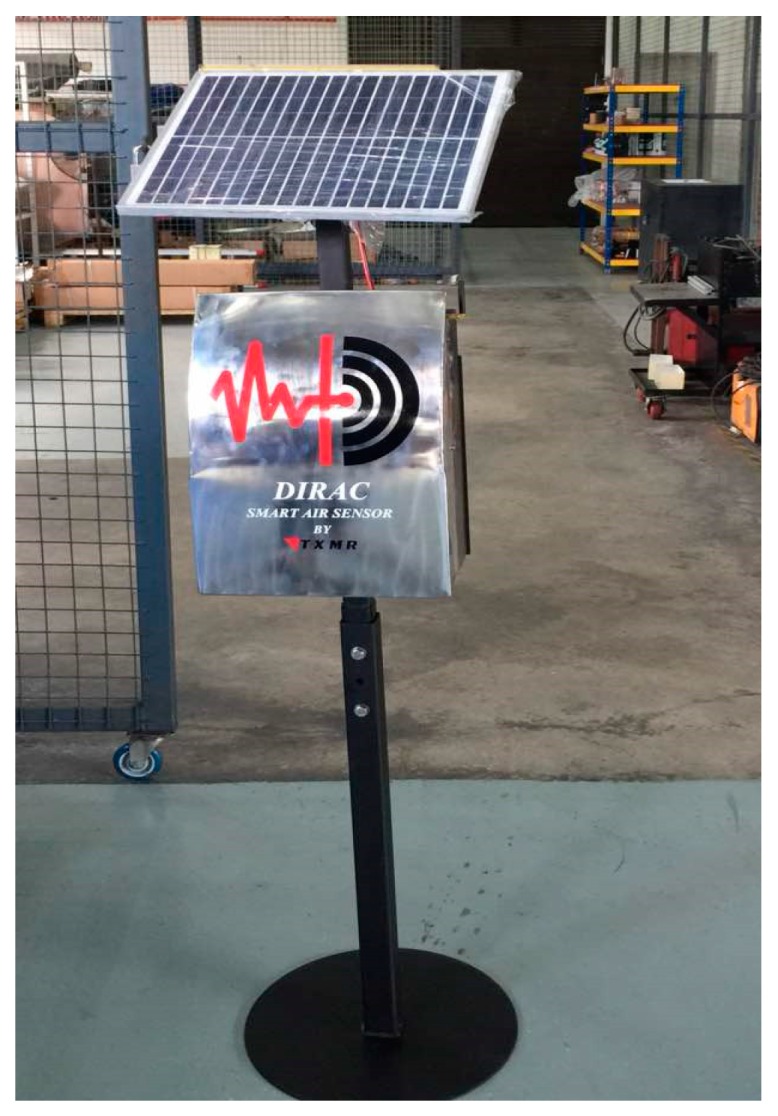
DiracSense.

The specific requirements for our sensing system were reliability and durability, that it is low-cost, portable, and easy to install by the user. Our system was designed to measure gas pollutants that are indexed by Malaysian ambient air quality standards at typical ambient concentrations. The result of our prototype development is DiracSense, as shown in Figure 1. DiracSense collects, analyzes and shares air quality data using wireless communication. Using the Internet of Things (IoT) scenario allows data to be sent remotely to a web server such as Google drive or Dropbox periodically as well as the visualization of numerical and graphical values over time. An Android mobile phone application was used to display the data to facilitate users in obtaining air quality data.

#### 2.1.2. Structure Design

**Figure 2:**
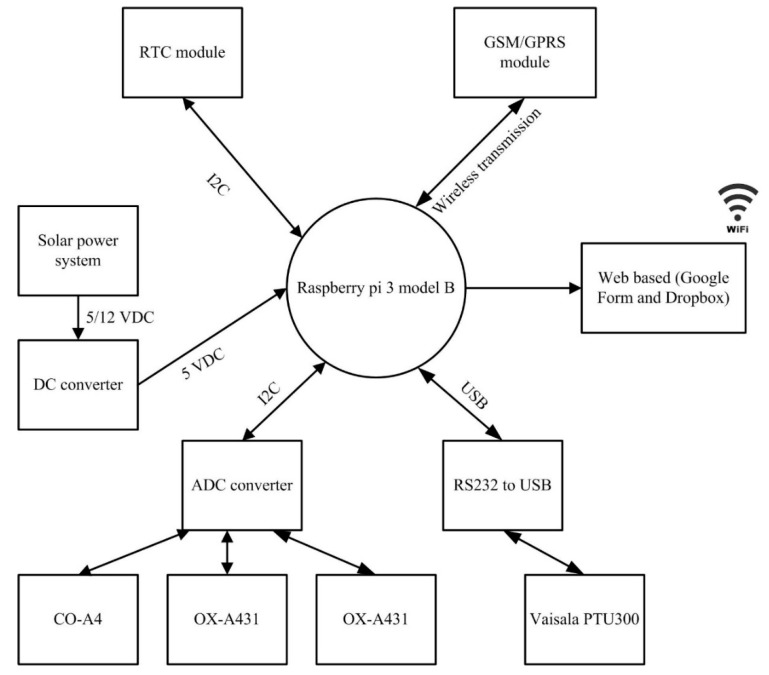
DiracSense system architecture.

The main part of DiracSense is in the form of a programmable system on a single board computer known as Raspberry Pi. This is the part of DiracSense which initializes all the software protocols required for the operation of the instrument. Looking at the structure of DiracSense (Figure 2), the component that was embedded can be divided into four parts: the sensing unit; the analogue digital converter (ADC) unit; the processing and storage unit; and the transmission unit and power supply.

The DiracSense has three 4-electrode electrochemical or EC sensors and one built-in and one external meteorological sensor manufactured by Alphasense (Alphasense Ltd., Great Notley, Braintree, UK) and Vaisala (Helsinki, Findland), respectively. The EC sensors are used for the measurement of gas pollutants such as CO, NO_2_ and O_3_, as shown in Table 1, while the meteorological parameters including pressure (P), temperature (T), and relative humidity (RH) are measured by a PTU300 sensor. The EC sensors used in our system were chosen such that they satisfy the concentration range and measurement accuracies required for ambient application while not compromising on the low power consumption requirements. The analogue output signals from the EC sensors are converted to digital signals via an onboard ADC before being fed to a single board computer. Signal/data processing, storage and transmission are also performed by the single board computer. Other hardware includes an 802.11 b/g/n wireless LAN and Bluetooth 4.1 used for the IoT. A 12 VDC battery integrated with solar power and DC/DC converter is used for the power supply unit. Figure 2 presents the design flowchart of the DiracSense, where the main components with the main measurement system are highlighted. 

#### 2.1.3. EC Sensors

The EC sensors are operated in amperometric mode, meaning the current resulting from the redox reaction is proportional to the concentration of the target gas. The current is measured using suitable electronics in a potentiostat configuration and has either a linear or logarithmic response [11]. Typically, the EC sensor consists of a cell made up of three electrodes which are separated with wetting filters. The filters are hydrophilic separators allowing the electrodes to come into contact with the electrolyte as well as allowing transport of the electrolyte through capillary action [13]. However, AlphaSense have developed an electrochemical sensor which consists of four electrodes designated working, reference, counter and auxiliary electrodes.

The sensing electrodes are the working and counter electrodes, both of which serve as sites of redox reaction. These electrodes are coated with selected high-surface-area catalyst materials that facilitate optimal reaction mechanisms as well as providing selectivity towards the target gas species. The oxidation/reduction reaction at the working electrode is balanced with a complementary reduction/oxidation reaction at the counter electrode (this is characteristic of a complete redox reaction) [13]. The redox pair results in the transfer of electrons (flow of current) between the working and counter electrodes. The reference electrode is used to stabilize and maintain the working electrode at a constant potential; this process ensures sensor response linearity over the range of uses. The auxiliary electrode (in a 4-electrode EC) is designed exactly like the working electrode except it is not in contact with the target gas species; as such it provides information on the effect of temperature on the overall recorded signal [15]. 

These sensors are declared to have lower detection limits and, power low power consumption. Other desired qualities include relatively fast response times (<20 s) and less sensitivity to changes in interfering gases and environmental conditions compared to other types of low-cost sensors such as metal-oxide-semiconductor (MOS) sensors. However, they are also larger in size and more expensive (<$100) [11,12].

### 2.2. Machine Learning

#### 2.2.1. Adaptive Neuro Fuzzy Inference System

An adaptive neuro fuzzy inference system (ANFIS) is a machine learning modeling technique introduced by Jang [26]. Its concept uses the intelligent hybrid method which integrates a neural network and fuzzy inference system (FIS). Typically, the ANFIS structure has functionality equivalent with the Takagi-Sugeno First-Order fuzzy model, wherein its construction is based on three conceptual frameworks including a rule base, a membership function (MF) and a fuzzy reasoning [20]. In a FIS the most difficult part is obtaining a MF and rule base. There is no procedural or protocol standard to construct these using the trial-and-error method. Thus, the capability of the neural network can be used to adjust these parameters. Using a self-learning algorithm, the parameters of the MF and rule base are adjusted in adaptive form. Fuzzy logic deals with its capability on the decision and uncertainties due to its structured knowledge-based representation. Neural networks are known for their self-organization and learning ability. Thus, an ANFIS has the advantages of both neural network and fuzzy logic capability. 

**Figure 3:**
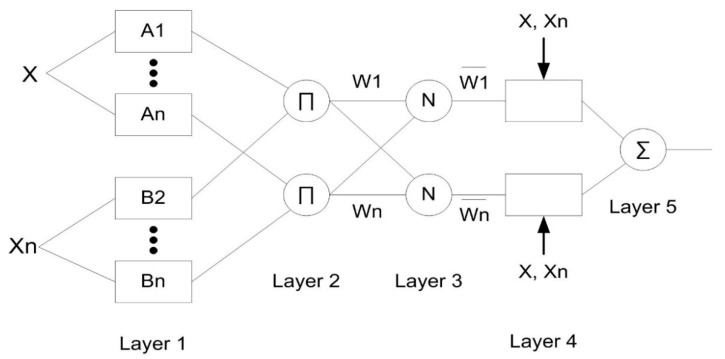
ANFIS model structure.

The ANFIS structure is related to the multilayer feed-forward network without weight in the network. The number of layers is fixed (about five layers) which represents the function of a fuzzy inference system, as shown in Figure 3. The first and fourth layers are made up of the fuzzy set parameter (called the premise parameter) and the linear parameter of rule (called the consequent parameter). These parameters can be adjusted using the learning algorithm to reduce the network error. The remaining layers are fixed parameters which contain the evaluation process.

Each layer has different functions; a brief explanation about the layers is as follows: The first layer contains a node which functions to produce a membership grade of the input layer using the MFs grade of the Fuzzy set. Usually, the gauss and generalized bell MFs are considered in this node. Every MF has parameter sets—called premise parameters—with the ability to change the shape of the MF. As the premise parameter values change, the shape of the MFs will vary accordingly. The second layer consists of a fixed node, where this node is multiplies all incoming signal across the entire node and evaluates it via an operator (T-norm operator) to obtain the signal output. This procedure is known as the firing strength of a rule. The third layer, or the normalized firing strength procedure, is a layer which consists of a calculation ratio for each firing strength with the sum of all firing strengths. The calculation of the network output is conducted with the linear equation formula by multiplying the normalized firing strengths and weight averages of each rule. These procedures are performed in the fourth and fifth layers. 

As mentioned, there are two key parameters (the premise and consequent parameters) which are adjustable in order to reduce the error of model. In this study, the hybrid learning algorithm introduced by Jang [26] is used to optimize these parameters. This algorithm is expanded from the combination of the gradient descent or backpropagation learning algorithms with the least squares estimates (LSE). There are two steps that should be performed in this learning algorithm. The first step is forward passing, where the consequent parameter value is updated by the LSE. At the same time, the premise parameters in the first layer are fixed-rate and then the error-rate will pass backward. On the other hand, the gradient descent is used during the second step to improve the premise parameters by minimizing the overall sum of the squared errors.

In order to optimize the number of MFs and the rule in the ANFIS model, fuzzy clustering is employed to generate MFs and the rule base automatically. Increasing the number of MFs on the network will affect the number of controlling rules and consequently computation can be time-consuming. Moreover, the fuzzy clustering method has the capability to avoid the uncertainty of data grouping [21]. With fuzzy clustering, the number of MFs and rules on the network is related to number of clusters that are generated. In this part, fuzzy subtractive clustering (FSC) is proposed as the fuzzy clustering method. FSC is very useful since as the number of clusters is not fixed it can automatically define the number of clusters based on the density of data points, which refers to the neighborhood radius value.

#### 2.2.2. Artificial Neural Network

**Figure 4:**
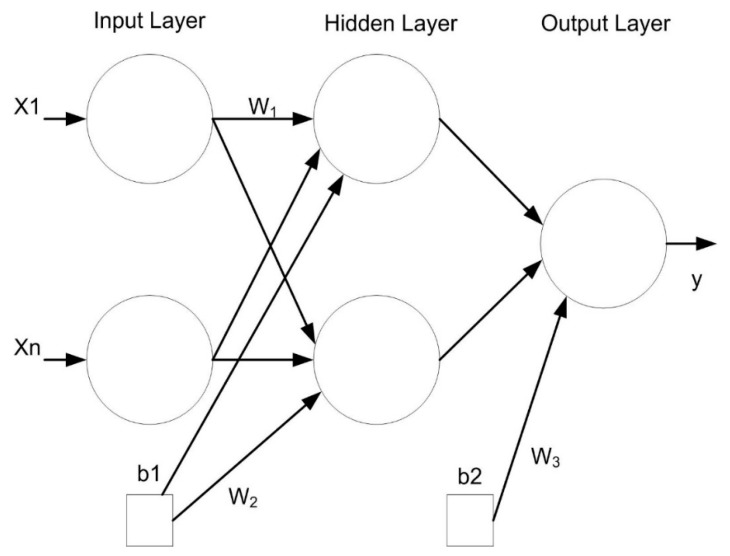
MLP model structure.

Artificial neural networks, known as ANNs, are popular modelling techniques that are frequently used in many applications related to scientific, engineering, medical, socio-economic, image processing and mathematical modelling [27]. A multi-layer perceptron (MLP) is a type of ANN structure and is used in this study. This structure has been frequently used to develop models which have very complex functions appropriate for the calibration of sensors. In general, the MLP has a feed-forward structure containing of input, hidden and output layers, as shown in Figure 4.

The work flow of the MLP is one direction, the information comes to the first layer (the input layer) before passing across the hidden layer and then to the last layer (the output layer). In addition, the structure of the MLP uses weighted sum of the input to produce the activation unit. This activation will be passed to the activation function to obtain the output in the output layer. By determining the number of layers and nodes in each layer, as well as the weights and thresholds of the network properly, it will minimize errors made by the network. This is part of the learning algorithm carrying out either supervised or unsupervised learning to optimize the result.

In this study the Levenberg-Marquardt learning algorithm, which is most commonly used for training [28], was employed to automatically adapt the value of weights and thresholds on the MLP network in order to minimize the output of error. The error of a specific configuration of the network can be found via test performances of all the training cases implemented on the network, then comparing the actual output generated by the network with the target or desired outputs. The differences of output units are calculated to give an error network value as a sum squared error, where the individual error of each layers is squared and summed at the same time. Further details about this algorithm can be found in [28]. 

### 2.3. Laboratory Calibration

As mentioned in Table 1, the variants of EC sensors used in this study are CO-AF, OX-AF and NO_2_-AF sensors for CO, O_3_ and NO_2_, respectively. These variants were designed to measure at low mixing ratios (units of parts per billion volume, ppb to few tens of parts per million volume, ppm range), achieved by the manufacturer improving both the sensitivity and sensor signal-to-noise ratio [13]. As part of the sensor performance tests, laboratory tests for all gases were conducted at the Center for Atmospheric Science, Chemistry Department, University of Cambridge (Cambridge, UK).

Concentrated gas standards supplied by Air Liquide UK Ltd. (Air Liquide UK Ltd., Wolverhampton, UK) and high-purity zero air were used to conduct laboratory testing of sensor performance at ppb mixing ratios. Zero air was generated using a Model 111 Zero Air Supply instrument (Thermo Fisher Scientific, Franklin, MA, USA). This is done by scrubbing ambient air of trace gas species such as CO, NO, NO_2_, O_3_, SO_2_ and hydrocarbons using a Purafil (Purafil Inc., Doraville, GA, USA) and catalytic purification system. The gas standard containing 20 (±2%) ppm NO, NO_2_, SO_2_ and 200 (±2%) ppm of CO in N_2_ was diluted to lower mixing ratios by mixing with zero air using the Thermo Scientific Model 146i Multi-Gas Calibrator which also produces the O_3_ used in the calibration procedure.

A two-point (zero and span) calibration was conducted for CO, NO, O_3_ and NO_2_ at a flow rate of 4 L/min. The target mixing ratios used were 1100 ppb (parts per billion by volume), 392 ppb, 425 ppb and 388 ppb for CO, NO, NO_2_ and O_3_, respectively. Calibration gas mixing ratios were typical of those expected to be present in the urban environment. These mixing ratios were confirmed using Thermo Scientific analyzers, models 48i, 42i and 49i for CO, NO/NO_2_ and O_3_ respectively. Test gases were delivered to the sensors using a special Teflon-based manifold to reduce the T_90_ acquisition time. Although the sampling time for each sensor was 1 s, data were averaged to give 10 s measurements.

The model provided by the sensor manufacturer to translate the voltage signal from the electrodes of EC sensor to mixing ratio is presented in Equation (1):

(1) $$Y=\;\frac{\left(\mathrm{WE}-{\mathrm{WE}}_{T}\right)-\left(\mathrm{AE}-{\mathrm{AE}}_{T}\right)}{S_{T}}.$$

where Y is the mixing ratio of target gas measured by EC sensor in units of ppb. WE (working electrode) and AE (auxiliary electrode) are the measured signal in millivolts (mV) for the two electrodes, WE_T_ and AE_T_ are the total zero offset of WE and AE (mV), respectively, corresponding to the signals recording during the zero-air calibration. The last parameter S_T_, is the total sensitivity of EC sensor (mV/ppb). WE_T_, AE_T_ and S_T_ were provided by the sensor manufacturer.

With these tests, we can assess the reliability of the calibration parameters (including the cross-interference information for Ox sensor) provided by the sensor manufacture and if necessary generate new correction factors.

### 2.4. Field Campaign

The measurement campaign was conducted at the research building complex, Universiti Kebangsaan Malaysia (UKM, Bangi, Malaysia) from 9–29 December 2017. This study only involved an O_3_ comparison as there were no other reference instrumentation at the study location. A model Serinius 10 UV photometric analyzer (EcoTech, Melbourne, Australia) was used in this campaign as the reference instrument for surface O_3_. This analyzer was well maintained and had been calibrated prior to this campaign. The calibration was performed based on a 7-point standard from low to high concentrations, with a range of interest of 0.1 to 200 ppb and detection limits of 0 to 50 ppb. During this campaign, DiracSense was located close to the inlet of the reference instrument to ensure good co-location.

One important step when developing a model using the soft computing technique is selecting the input variable and the size of the training data set, as they have capability to influence the determination of the network and the strength of model [24]. Generally, the training data are used as a knowledge base and the rules of the model in order to catch all characteristics of the target. Two other types of data are also used in this developing stage, including the validation and testing data. The validation data are used to make certain that the model is trained to have the capability to generalize training data in order to represent the target data to avoid overfitting during the training period. The overfitting will occur if the model is trained too much, causing the model to lose its ability to generalize and adjust any data that was not included in training process [20]. At the same time, the testing data are used to check the performance capabilities of the resulting model. 

In this study, two source data sets (raw data from the EC sensor and the reference instrument) were applied in the developing model as input and target data, respectively. All data should be equal interval resolution data. Since the data from the O_3_ reference instrument had a time resolution of 10 min, 10-min averaged raw data from DiracSense were used in order to equal the resolution of the data. All data collected were divided into three data subsets (training, validation and testing). The training data set was taken from 9–13 and 18–22 December, while the validation data spanned 14–17 December 2017. The remaining seven days (23–29 December 2017) were used for the testing data sets (see Table 2). The cross-validation method was used to divide data into these three subsets. All the data sets during the training period were quality controlled to exclude invalid or noise data flagged in the recorded raw data as Not a Number (NaN).

The configuration input models used for the calibration model are shown in the Table 3. The configuration input model consisted of working and auxiliary electrodes raw data (WE OX, and AE OX), NO_2_ gas and T data from the OX-A431 EC sensor, NO_2_-A43F EC sensor, and temperature sensor respectively. These configurations were proposed to avoid the limitation of data availability and to investigate the impact of different combinations of the variables on the correction of EC sensor. Six combinations of the input variables (see Table 3) were studied to investigate their effects on producing calibrated data from the EC sensor. The combinations make use of either two or four inputs. The choice of input variables in developing a calibration model is very important task as it can affect the performance of the model. Similarly, the size of the training data set is equally very vital, as it will affect the ability of the model in capturing all the characteristics of the desired output. In this study, we employed several statistical analysis techniques to evaluate the performance of the calibration model. These included the calculation of the coefficient correlation (r), percent error (PE) and root mean squared error (RMSE).

## 3. Results

### 3.1. Laboratory Test

#### 3.1.1. Response Time

**Figure 5:**
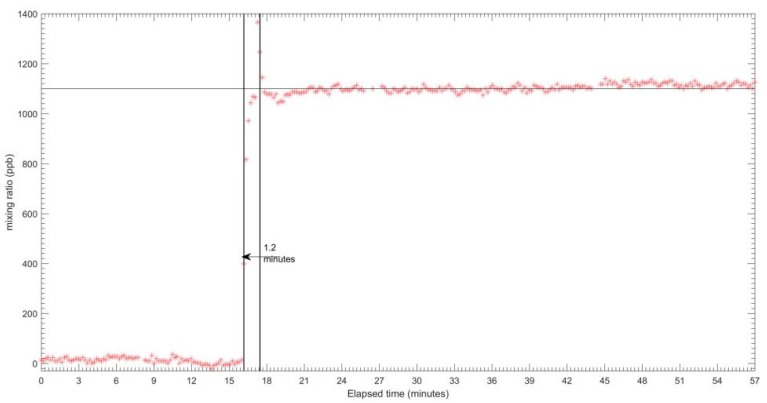
Response times of CO-A4 EC sensor from zero target gas to target gas.

**Figure 6:**
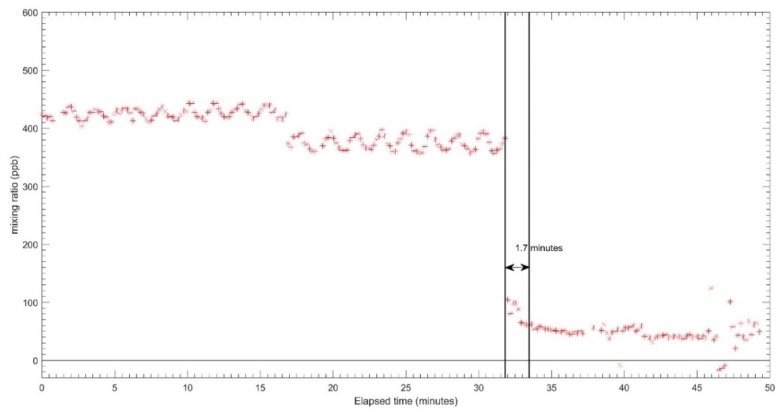
Response time of OX-A431 sensor from target gas to zero.

**Figure 7:**
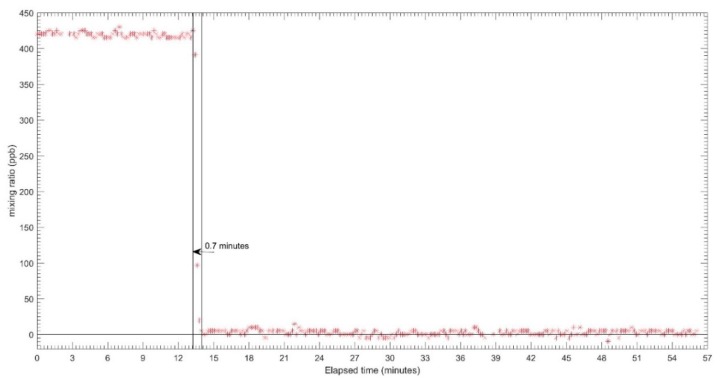
Response time of NO_2_-A43F sensor from target gas to zero.

The results for the response time tests for each sensor are shown in Figure 5, Figure 6 and Figure 7 for CO, O_3_ and NO_2_ respectively. The zero air and span gas standards were used to conduct this test. For this test, the different treatments were carried out for each sensor due to a technical issue during the testing process. For the CO EC sensor, the treatment performed was the rise response, while the others two sensors (OX and NO_2_ EC sensors) were treated with fall response. During the response time test, the CO EC sensor response time from zero to 90% of the full-scale target concentration was around 1.2 min, as shown in Figure 5. On the other hand, a response time of around 1.6 min was observed for the OX EC sensor in terms of falling response toward the zero gas points (Figure 6). The NO_2_ EC response time is shown in Figure 7, and these correspond to 0.7 min for target concentration to zero point. Moreover, the lag time was in the order of 5–20 s for all sensors for changing the concentration to the sensor reaching 10% of the desired gas point. 

#### 3.1.2. Laboratory Performance

**Figure 8:**
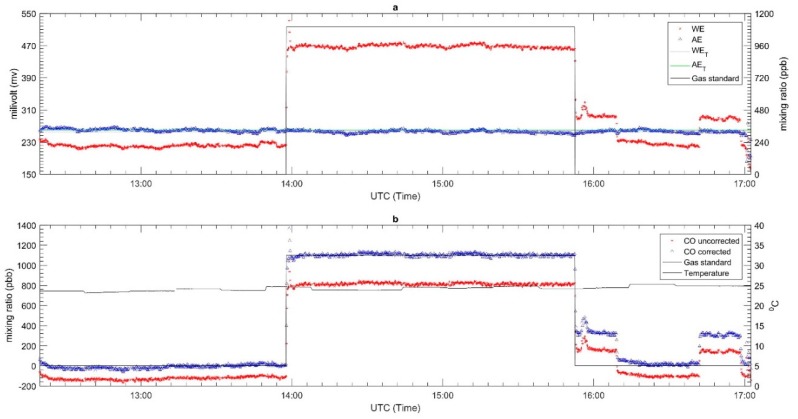
The laboratory calibration of CO-A4 EC sensor (**a**) the electrode signal along with the gas standard and (**b**) The signal converted to mixing ratio and temperature.

**Figure 9:**
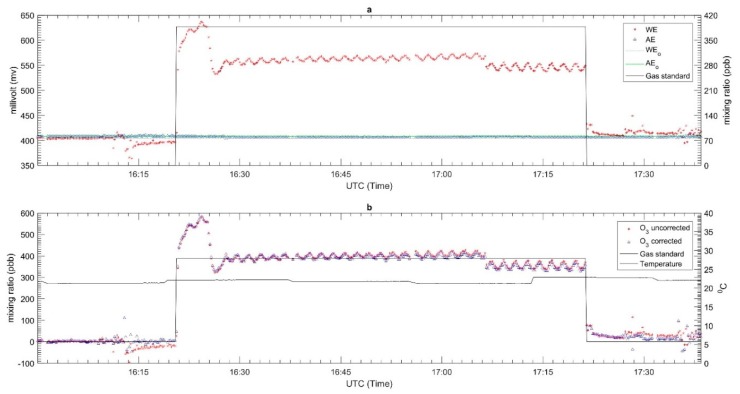
The laboratory calibration of OX-A431 EC sensor (**a**) the electrode signal along with the gas standard and (**b**) The signal converted to mixing ratio and temperature.

**Figure 10:**
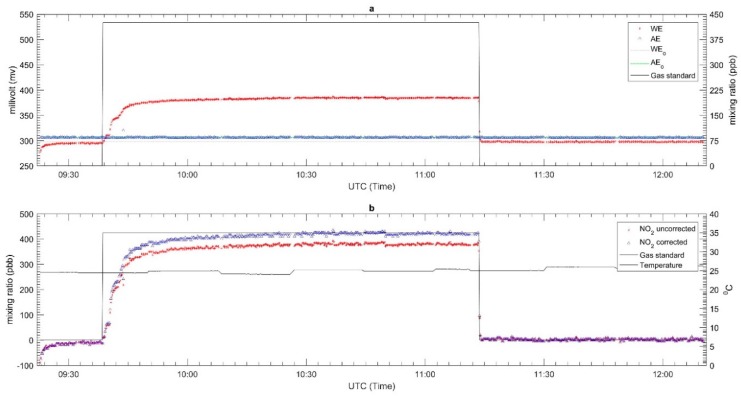
The laboratory calibration of NO_2_-A43F EC sensor (**a**) the electrode signal along with the gas standard and (**b**) The signal converted to mixing ratio and temperature.

The laboratory performance results for each sensor are described in Figure 8, Figure 9 and Figure 10 for CO, O_3_ and NO_2_, respectively. The top panel in each Figure corresponds to the plot of the WE and AE along with the gas standard mixing ratios. The red, blue, grey, green and black lines represent the WE, AE, WE_T_, AE_T_ and the two-point gas standards (span and zero). All WE signals from each EC sensor gradually increased once the span gas standard started flowing through the sensor and gradually decreased when the flow was switched to zero air. On the other hand, the AE signals remained relatively constant throughout the test period. The AE was not affected by the target gas as it is not in contact with it; its main function is to replicate the impact (if any) of temperature on the signal of the WE. Since these tests were carried out under controlled conditions with fairly constant temperature (see panel b in Figure 8, Figure 9 and Figure 10), this pattern of behavior is expected for the AE signals. In this case the zero point was chosen from the beginning and end of the experiment to know the drift signal from WE and AE when exposed to the zero air. The drift signal from WE and AE were computed from the actual output of WE and AE signals during zero air exposure compared with the total zero offset value of WE and AE from the factory calibration. For the CO sensor, it was found to have an insignificant drift in zero air—roughly −28 mv and −4 mv for WE and AE, respectively. Similarly, the NO_2_ and OX sensors did not have much drift; the WE and AE signals in both sensors only drifted around −1 to −2 mV.

The second panels of Figure 8, Figure 9 and Figure 10 present the converted signal in mixing ratio format based on Equation (1) and temperature. The mixing ratio derived from the EC sensors for both the uncorrected and corrected as well as two-point gas standard readings and temperature are in red, blue and black lines, respectively. The results showed that the mixing ratio for the uncorrected data do not match the span gas standard. For example, the uncorrected mixing ratio of CO had different readings of 287 ppb and −107 ppb respectively during when the span (1100 ppb) and zero gas standard were flowed across the sensor. Similar patterns were observed for the NO_2_ and OX sensor. For the NO_2_ sensor, the differences between the uncorrected and standard gas mixing ratios were about 67 ppb and −2 ppb for the span and zero respectively, while the for the OX sensor they were roughly −10 ppb and −8.1 ppb, respectively. Unlike Figure 8 and Figure 10, Figure 9 shows a ‘ripple’ signal during the injection gas standard. This might occur due to the mass flow control (mfc) which is used to generate gas trying to maintain a steady concentration. 

Since the calibration results shows large differences with the gas standard, we recalibrated the raw data using the results of this experiment. Equation (1) was modified by replacing the factory sensitivities with the new sensitivity values obtained from the new laboratory tests. The new sensitivity values were calculating by the determination of the peak (h_diff_) and lowest (l_diff_) difference values between WE and AE signals during the calibration procedure and dividing them with the span gas standard, as described in Equation (2). An offset was also included in the new expression as described in Equation (3). It determines the lowest difference value between WE and AE signal divided by the new sensitivity. Table 4 shows the offset and new sensitivity values for each sensor. The results of the recalibrated data set are presented in the second panels of Figure 8, Figure 9 and Figure 10.

(2) $$S_{T}=\frac{h_{\mathrm{diff}}-l_{\mathrm{diff}}}{\mathrm{ref}}$$

(3) $$\begin{array}{c} Y=\left(\frac{\left(\mathrm{WE}-{\mathrm{WE}}_{T}\right)-\left(\mathrm{AE}-{\mathrm{AE}}_{T}\right)}{S_{T}}\right)-\mathrm{offset}\\ \mathrm{offset}=\;\frac{l_{\mathrm{diff}}}{s_{T}} \end{array}$$

The OX sensor responds to O_3_ and NO_2_ in similar magnitudes according to factory calibration, but for the purposes of our study we want to use the sensor for O_3_ detection. We therefore determined the cross-sensitivity (fraction) to NO_2_ as part of the gas calibration tests. Based on the calibration result, we found that the cross-sensitivity value of the OX sensor to NO_2_ was about 0.74. The new expression for O_3_ based on Equation (2) is given by Equation (3), where S_T_ is OX sensitivity to O_3_, and NO_2_con is the recalibrated NO_2_ data using Equation (2):

(4) $$Y=\left(\left(\frac{\left(\mathrm{WE}-{\mathrm{WE}}_{T}\right)-\left(\mathrm{AE}-{\mathrm{AE}}_{T}\right)}{S_{T}}\right)-\mathrm{offset}\right)-\left(0.74*{\mathrm{NO}}_{2}\mathrm{con}\right)$$

### 3.2. Evaluation of Field Calibration

Following the laboratory tests, the sensor box was deployed in the field to assess the ambient performance of the device. Data from this deployment were compared to O_3_ reference measurements since this was the only reference data available at the comparison site (see Section 2.4).

**Figure 11:**
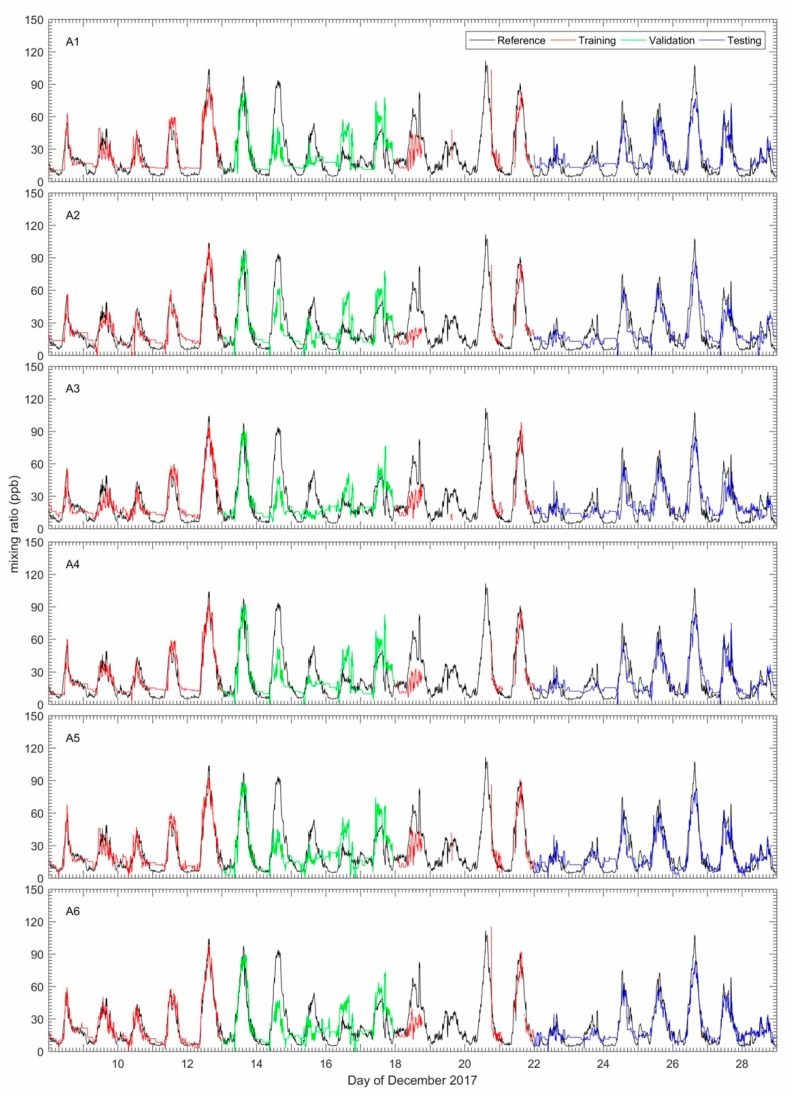
Variation of O_3_ measured from ANFIS calibration model compared with reference instrument for whole combination input.

In this work, as mentioned in Section 2.2.1, the FIS that implemented the ANFIS structure was developed automatically using the fuzzy subtractive cluster (FSC) and trained using the hybrid learning algorithm in order to achieve a satisfactory calibration model. The model performance was evaluated using several statistical methods such as mean absolute error (MAE), root mean square error (RMSE) and Pearson’s correlation coefficient (r), for the three categories of data (training, testing and validation datasets). Figure 11 shows the result of the calibration for O_3_ using the ANFIS method compared to the reference measurements during the training, testing and validation periods for whole the combination inputs described in Section 2.4. The black, red, green and blue dots shown in Figure 11 represent the reference, training, validation and testing data sets. The gaps in the time series seen in the figures correspond to periods where there were no data recorded by the EC sensor or temperature sensor. For the whole result, the calibrated O_3_ data using the ANFIS method shows good agreement (0.8 < r < 0.95) with the reference O_3_ measurements.

To achieve the best results from the ANFIS technique, different number epochs or iterations should be used during the training process in order to reach the minimum error given from the model. In addition, the validation data sets were also employed during the training period to avoid occurrences of overfitting [19]. As mentioned in Section 2.4, overfitting will occur if the output of a trained ANFIS model cannot replicate any other new input data not used for training or if the error is larger compared with when the new data is included in the training process. In our tests, 30% and 70% were allocated to the validation and training data sets, respectively. Moreover, a higher epoch or iteration during the training process results in improved performance of the model provided there is no overfitting.

**Figure 12:**
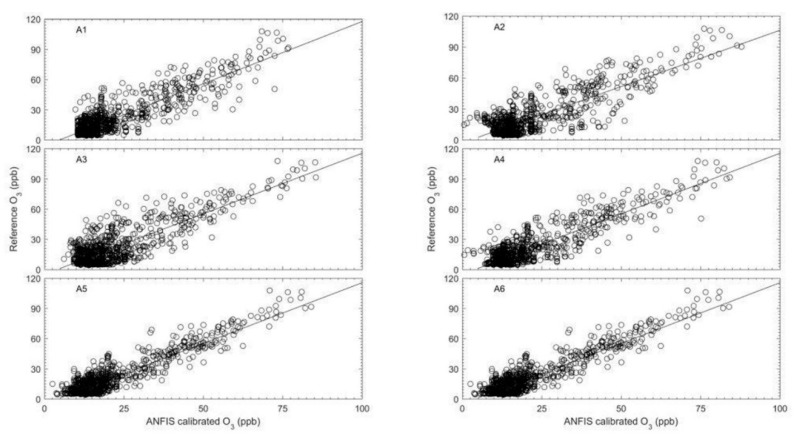
Scatter plot of O_3_ for the ANFIS calibration model and the reference instrument for the testing periods in Figure 11.

From the comparison of the different input combinations for the model, the four-input (A6) ANFIS model (WE OX, AE OX, T, NO_2_) showed the best agreement with the reference (bottom right panel of Figure 11, and highlighted entries in Table 5). The next best input combination was the 3-input (A5) ANFIS model (WE OX, AE OX, NO_2_) (with R ~ 0.90). Figure 12 shows the scatter plots for the different combinations in Figure 11. This figure presents the relative size of the bias and random error for each input combination for the testing period. The plot shows that all the different input combinations had good results, where the all data points fall close to the trend line. Nevertheless, the calibration model of O_3_ without WE OX in the combination input generated outputs with poor agreement with the reference O_3_ measurements. Although the WE OX is a key variable input in the calibration model process, the other variable inputs are also important in order to improve the accuracy of the calibration model. This is evident in the results as the accuracy of calibration model increased with more input variables. In addition, after the training period, four rules and membership functions were found to be the best combination for the ANFIS structure in terms of use for the four input combinations. Increasing the rules and MFs in the ANFIS structure can lead to an increase or decrease in model accuracy. This could be because the ANFIS structure becomes more complex as rules and MFs are increased to a certain point error in the model. The errors associated with the model gradually increase the more complex the ANFIS structure is becomes. Consequently, it would lead to model taking too much time to get the minimum target error.

The summaries of statistical comparisons of calibration models using the ANFIS technique on the OX EC with the O_3_ measured using the routine analyzer model are shown in Table 5. Increasing the number of input variables in the combination improved the accuracy and performance of the calibration model, as shown in the statistical analysis of Table 5. Comparing all possible combination inputs, the four-input combination (A6) had the lowest error with MAE and RMSE values of 6.431 ppb and 8.140 ppb respectively (Table 5). This demonstrates the viability of the ANFIS four-input model in producing calibrated O_3_ measurements. The results also showed that the accuracy and performance of the calibration model improved when the surface temperature and AE OX are selected rather than NO_2_. It can be seen from Table 5 that the combination input of A5 gives the second-lowest error.

### 3.3. Comparison of Calibration Models 

Besides the calibration model which was developed using the ANFIS model, two other calibration models were used to translate the signal from the EC sensor into the mixing ratio of O_3_. The first model was constructed with the MLP and the other used Equation (3) (Section 3.1.2). The calibration model based on the MLP was trained and tested using the same data sets as was used for the ANFIS model. The combination input A6 was chosen as the input network of MLP. In this case, diverse numbers of combination neurons in the hidden layer of network structure were tested to find out the best fit selection for this network. A feed forward network containing four input layers, seven hidden layers, and one output layer (4-7-1) with training using the Levenberg-Marquardt learning algorithm was used for this alternative calibration model. A tangent and linear sigmoid were used as activation transfer functions for the hidden layer and output layer of the network respectively.

**Figure 13:**
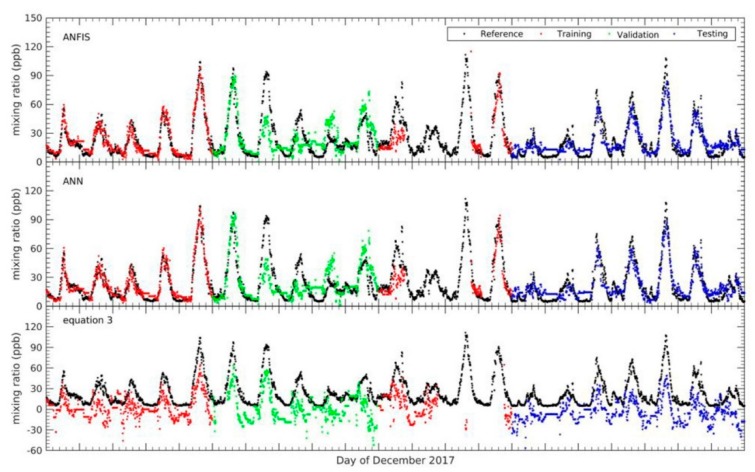
Comparison of O_3_ values obtained from the reference instrument and the three calibration models ANFIS, ANN (MLP), and Equation (3) during the training, validation and testing.

The comparison of the results of all models (ANFIS, MLP, and Equation (3)) are shown in Figure 13 during the training, testing, and validation periods. All three calibration models translated the raw OX signal into realistic mixing ratios of O_3_. However, the result for the model based on Equation (3) had larger errors and quite high amplitude to amplitude differences of about the 30 ppb when compared to the reference measurements. The output from Equation (3) did not always follow the reference pattern and it had a systematic negative zero offset. On the other hand, the MLP model result was similar to the ANFIS model. It can be seen from the pattern of mixing ratios that it almost completely followed the mixing ratio pattern given by the reference instrument.

**Figure 14:**
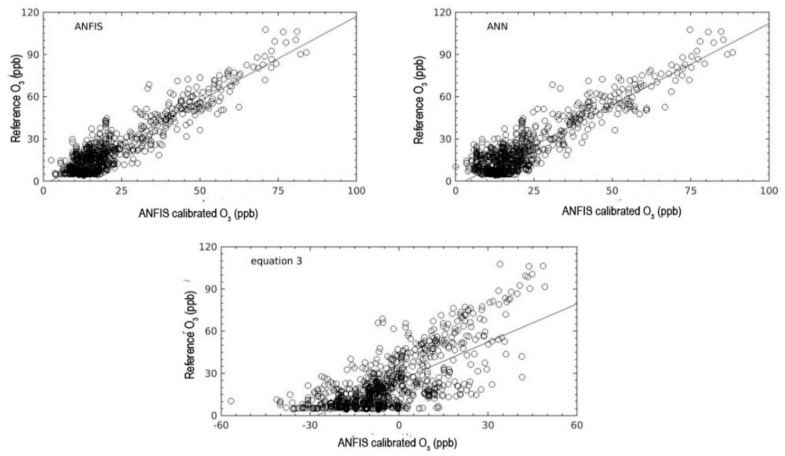
The relationship between O_3_ values measured with the reference instrument and the three calibration models ANFIS, ANN (MLP) and Equation (3) during the testing period.

Figure 14 shows the scatter plot for the comparison of the three models with the reference O_3_ measurements for the testing period. As described in Figure 14, the calibrations of the ANFIS and MLP models had good agreement and a consistent relationship with the reference instrument. Almost all the data points for both models track the trend line in Figure 14. In contrast, Equation (3) data points are more scattered and distant from the trend line. The ANFIS and MLP calibration models had almost the same values in terms of r value which were larger than 0.90 (see Table 6). This indicates that the MLP network can be used as an alternative model for calibrating O_3_ measurements from an EC sensor to improve accuracy and performance. This is in accordance with other studies. For example, Borrego et al. [29] examined a feed forward neural network (FFNN) as a calibration model to improve the accuracy and performance of an EC O_3_ sensor. With a different strategy on the neural network structure and different data sets, the model developed obtained promising results with r in the range 0.89 to 0.92 (see Table 7). 

Statistical analysis from all calibration models are presented in Table 6. From Table 6, it found that during the validation period the calibration model constructed with ANFIS had the lowest error of 8.140 ppb and 6.431 ppb for RMSE and MAE, respectively, relative to the other two models. However, the MLP network was found to have the lowest error during the training process. This indicates the ANFIS is more capable of capturing the data sets that are not part of the training data compared to the other two models. The calibration model using Equation (3) showed the biggest error for all data sets, including training, validation and testing.

## 4. Conclusions

Three EC sensors from AlphaSense were constructed to measure CO, NO_2_, and O_3_. The sensors behaved highly linearly in laboratory experiments and had response times of around 0.5–1.6 min. During the laboratory experiment, a simple equation was used to translate the signal to mixing ratio and was calibrated by adding a correction in order to achieve the minimum difference against the gas standard. We found that with the added corrections such as the new sensitivity and offset to the equation, the difference values between mixing ratio of EC sensor and gas standard became decreased. Furthermore, this equation is deployed together with the other calibration model which constructed using the machine learning to translate signal to mixing ratios in the field experiment.

After the laboratory experiment was performed, field tests were undertaken to investigate the performance of the EC sensor when measuring gases in ambient conditions. However, due to the lack of other routine instruments at the UKM, the field calibration for the EC sensor focused on the mixing ratio of O_3_. Several calibration models were constructed in order to improve the accuracy and performance of the EC sensor, including ANFIS, MLP and the simple equation which was calibrated during the laboratory experiment. This study has successfully demonstrated the capability of calibration models constructed using the ANFIS technique to improve the accuracy and performance of EC sensors in order to measure mixing ratios of O_3_. Since the input parameter is one of the crucial components in developing a machine learning, few combination inputs were evaluated to obtain the best structure input network for the ANFIS in order to obtain high accuracy. This is parallel with the other studies, wherein the correctly input selection as well as it has correlated with the target output interest will be affected on the performance of model, especially in the local minima problem [18,19]. From the configuration input combination, we found that the combination input with only the mixing ratio of NO_2_ was not able to translate the output signal of the EC sensor to the mixing ratio of O_3_ correctly. However, adding new input variables such as WE OX, AE OX and T will gradually improve the calibration model developed using ANFIS. The combination of input variables containing WE OX, AE OX, T, and NO_2_ were the best selection in terms of configuration input network for the calibration model. Moreover, the input combination containing WE OX, AE OX and T can be an optional configuration input network when mixing ratio of NO_2_ data are limited. 

Other models were tested, and the results demonstrated that the calibration model constructed using MLP has potential as a competitive model to ANFIS. It had the second-strongest coefficient correlation. However, the calibration model using the ANFIS technique still outperformed the other models in terms of the statistical evaluation criteria. The calibration model constructed using ANFIS had the lowest RMSE and MAE values as well as the highest correlation coefficient during the validation period.

Nevertheless, it should be noted that the representatives of measurements in this result only showed during the conditions of this campaign. The result may be different for longer time periods, since the model experiment was only conducted for seven days during the validation period. Moreover, this calibration model should be regarded as the on-site calibration since it required ground-based data as the target data for the area of deployment. It very hard to claim this model can be used as a generalized calibration model since the environments of polluted areas have different variations. In addition, the ageing of sensors is required to be considered since it leads to decreasing sensor performance [29]. However, this model can be deployed in other areas of interest if the conditions are similar or the process would be adding more knowledge into the model.

Regardless, the calibration model constructed using machine learning including ANFIS and MLP has a usable ability to improve the accuracy and performance of EC sensors in terms of measuring mixing ratios of O_3_. It will beneficial for researchers and communities who want to measure the O_3_ in the air for air quality monitoring purposes using the low-cost sensor since the results showed that it was in close agreement with the reference instrument. 

## Figures and Tables

**Table 1:** List sensor selection for different gases.

Sensor Type	Measured Gas	Sensitivity (mv/ppb)
NO_2_-A43F	NO_2_	0.229
OX-A431	O_3_	0.401
CO-A4	CO	0.267

**Table 2:** List parameters which used for the training, testing and validation during the field calibration.

Process	Variable	Date	Data Points	Number of Days
Training	Raw data from OX-A431 sensor NO_2_ mixing ratio from NO_2_-A43F sensor Temperature	9–13 and 18–22 December 2017	1440	9
Validation	Raw data from OX-A431 sensor NO_2_ mixing ratio from NO_2_-A43F sensor Temperature	14–17 December 2017	576	4
Testing	Raw data from OX-A431 sensor NO_2_ mixing ratio from NO_2_-A43F sensor Temperature	23–29 December 2017	1008	7

**Table 3:** Summary of the configuration input model.

Combination	Input	Output (Pollutant)
A1	WE OX(t), AE OX(t)	O_3_
A2	WE OX(t), T(t)	O_3_
A3	WE OX(t), NO_2_(t)	O_3_
A4	WE OX(t), AE OX(t), T(t)	O_3_
A5	WE OX(t), AE OX(t), NO_2_(t)	O_3_
A6	WE OX(t), AE OX(t), T(t), NO_2_(t)	O_3_

**Table 4:** Summary the new sensitivity and offset of each EC sensor.

Sensor	Sensitivity (mv/ppb)	Offset (ppb)
NO_2_-A43F	0.207	−4.829
OX-A431	0.415	−2.41
CO-A4	0.226	−71.420

**Table 5:** Statistical comparison between O_3_ measurements from reference instrument and EC sensor OX signal calibrated using the ANFIS technique. Results are presented for the training, validation and testing periods.

Period	Input	R	RMSE (ppb)	MAE (ppb)
Training	A1	0.873	10.066	6.859
	A2	0.911	8.0961	6.453
	A3	0.837	11.286	7.256
	A4	0.914	7.967	6.331
	A5	0.889	9.453	6.081
	A6	0.945	6.395	4.718
Validation	A1	0.908	8.153	6.253
	A2	0.919	7.887	6.178
	A3	0.900	8.484	6.947
	A4	0.933	7.027	5.362
	A5	0.920	7.260	6.050
	A6	0.939	6.746	5.224
Testing	A1	0.873	9.855	7.831
	A2	0.830	10.786	8.741
	A3	0.836	10.867	8.867
	A4	0.880	9.463	7.522
	A5	0.901	8.804	6.842
	A6	0.922	8.140	6.431

**Table 6:** Different errors in translating signal to mixing ratios of O_3_ using the calibration models which were developed using ANFIS, MLP and Equation (3) as compared with the mixing ratio of O_3_ obtained from the reference instrument during the training, validation, and testing period.

Period	Calibration Model	r	RMSE (ppb)	MAE (ppb)
Training	ANFIS	0.945	6.395	4.718
	MLP	0.955	5.815	4.491
	Equation (4)	0.636	27.495	22.814
Validation	ANFIS	0.939	6.746	5.224
	MLP	0.941	6.638	5.089
	Equation (4)	0.642	28.653	24.643
Testing	ANFIS	0.922	8.140	6.431
	MLP	0.904	8.505	7.034
	Equation4	0.715	28.931	25.832

**Table 7:** Comparison calibration model for EC O_3_ sensor with previous study.

Authors	Machine Learning	Network Structure	Gas Measured	r
This study	ANFIS (10 min)	Four membership function and rules	O_3_	0.922
	MLP (10 min)	Seven hidden and one output layers with tangent and linear sigmoid activation function	O_3_	0.904
Borrego et al. [29]	FNN (1 h)	Single hidden and one output layer with sigmoid activation function	O_3_	0.89–0.92
	FNN (1 min)	Five hidden and one output layer	O_3_	0.93
